# Morphological and molecular comparison of HIV-associated and sporadic inclusion body myositis

**DOI:** 10.1007/s00415-023-11779-y

**Published:** 2023-06-06

**Authors:** Sinja Vogt, Felix Kleefeld, Corinna Preusse, Gabriele Arendt, Stefan Bieneck, Anna Brunn, Martina Deckert, Benjamin Englert, Hans-Hilmar Goebel, Anja Masuhr, Eva Neuen-Jacob, Cornelia Kornblum, Jens Reimann, Federica Montagnese, Benedikt Schoser, Werner Stenzel, Katrin Hahn

**Affiliations:** 1 Department of Neurology, Charité, Universitätsmedizin Berlin, Corporate Member of Freie Universität Berlin and Humboldt-Universität zu Berlin, 10117 Berlin, Germany; 2 Department of Neuropathology, Charité, Universitätsmedizin Berlin, Corporate Member of Freie Universität Berlin and Humboldt-Universität zu Berlin, 10117 Berlin, Germany; 3 BIH Charité Clinician Scientist Program, BIH Biomedical Innovation Academy, Berlin Institute of Health at Charité, Universitätsmedizin Berlin, 10117 Berlin, Germany; 4 Neuro-Centrum Düsseldorf, 40211 Düsseldorf, Germany; 5 Department of Internal Medicine, Rheumatology, Schlosspark-Klinik, 14059 Berlin, Germany; 6 Faculty of Medicine, Institute of Neuropathology, University Hospital Cologne, 50937 Cologne, Germany; 7 Department of Internal Medicine, Infectiology, Auguste-Viktoria-Klinikum, 12157 Berlin, Germany; 8 Department of Neuropathology, University Hospital Düsseldorf, 40225 Düsseldorf, Germany; 9 Department of Neurology, University Hospital Bonn, 53127 Bonn, Germany; 10 Department of Neurology, Friedrich-Baur-Institute, University Hospital Munich, 80336 Munich, Germany; 11 Center for Neuropathology and Prion Research, Ludwig-Maximilians University Munich, 81337 Munich, Germany

**Keywords:** Inclusion Body Myositis, KLRG1, HIV-IBM, IBM-SD

## Abstract

**Objective:**

The molecular characteristics of sporadic inclusion body myositis (sIBM) have been intensively studied, and specific patterns on the cellular, protein and RNA level have emerged. However, these characteristics have not been studied in the context of HIV-associated IBM (HIV-IBM). In this study, we compared clinical, histopathological, and transcriptomic patterns of sIBM and HIV-IBM.

**Methods:**

In this cross-sectional study, we compared patients with HIV-IBM and sIBM based on clinical and morphological features as well as gene expression levels of specific T-cell markers in skeletal muscle biopsy samples. Non-disease individuals served as controls (NDC). Cell counts for immunohistochemistry and gene expression profiles for quantitative PCR were used as primary outcomes.

**Results:**

14 muscle biopsy samples (7 HIV-IBM, 7 sIBM) of patients and 6 biopsy samples from NDC were included. Clinically, HIV-IBM patients showed a significantly lower age of onset and a shorter period between symptom onset and muscle biopsy. Histomorphologically, HIV-IBM patients showed no KLRG1^+^ or CD57^+^ cells, while the number of PD1^+^ cells did not differ significantly between the two groups. All markers were shown to be significantly upregulated at gene expression level with no significant difference between the IBM subgroups.

**Conclusion:**

Despite HIV-IBM and sIBM sharing important clinical, histopathological, and transcriptomic signatures, the presence of KLRG1^+^ cells discriminated sIBM from HIV-IBM. This may be explained by longer disease duration and subsequent T-cell stimulation in sIBM. Thus, the presence of TEMRA cells is characteristic for sIBM, but not a prerequisite for the development of IBM in HIV^+^ patients.

**Supplementary Information:**

The online version contains supplementary material available at 10.1007/s00415-023-11779-y.

## Introduction

HIV-associated myositis has been described as a complication of HIV/AIDS since the beginning of the HIV/AIDS pandemic [1]. Different subtypes of myositis can occur in the context of HIV infection and may be classified into the following groups: isolated mitochondrial abnormalities (IMA), polymyositis (PM), inclusion body myositis (IBM), Immune-mediated necrotizing myopathy (IMNM) and non-specific myositis [2]. HIV-infected patients who develop IBM have been shown to differ from sporadic IBM (sIBM) patients in terms of earlier onset of illness and, in some cases, better response to immunomodulatory treatment [3, 4]. SIBM is the most common idiopathic inflammatory myopathy (IIM) above the age of 50 years, with an estimated prevalence of 35/1 million [5]. Based on molecular data, the concept of an IBM spectrum disease (IBM-SD) has recently been introduced [6]. IBM-SD describes the clinical and histomorphological spectrum ranging from mild inflammation and mitochondrial abnormalities to full-blown IBM [6]. The pathophysiology of both sIBM and HIV-IBM is still incompletely understood. In the context of HIV, despite antiretroviral therapy (ART) and in many cases normal numbers of total CD4^+^ cells and a suppressed viral load, complex immunological alterations persist. A lower proportion of naïve CD4 and CD8 T cells and more effector memory T cells have been described in HIV patients compared to HIV-seronegative patients in the same age group [7, 8]. Similar changes in the immune system have been detected in elderly, otherwise healthy individuals as well [9, 10]. This has been assumed to be related to accelerated ageing of immune processes in patients with HIV [7, 8]. In addition, HIV infection causes chronic viral antigen stimulation, leading to T cell exhaustion. In this process, T cells functionally deteriorate and upregulate the expression of PD1 [11, 12]. A correlation between functional impairment of CD8 T cells, high viral load and low CD4 T cell counts was described [11], and T cell exhaustion was shown to be partially reversible under ART with a decrease of PD1 expression on HIV-specific CD8 T cells [11]. Hence the functionality of HIV-specific T cells improves with antiretroviral therapy, but systemic PD1 expression levels remain higher compared to seronegative individuals [11]. In sIBM, so-called exhausted T-cells with numerous PD1^+^ cells were detected in the muscle [13, 14]. These cells are characterized by a cytotoxic signature (granzyme A, B, H, K, perforin) and a highly differentiated T cell signature (KLRG1^+^, CD244^+^, T-bet^+^, CD57^+^/CD28^−^, CD62L^−^) [15]. During the differentiation process into an effector memory population, T cells lose markers such as CD27, CD28 and CCR7 and gain CD57 and KLRG1, the latter being a specific marker for this population [16]. These cells are called “Terminally differentiated effector memory T cells” (TEMRA) [17] and are further characterized by a limited proliferative capacity and strong effector functions [12]. A recent study found 79% of CD8 T cells positive for KLRG1 and 48% positive for CD57 at the surface of muscle fibres in IBM patients [18]. Additionally, it was shown that the proportion of KLRG1-positive cells in the blood increases with disease progression [19]. In summary, KLRG1 has become both an important diagnostic marker as well as a possible therapeutic target in the context of sIBM [20]. Of note, the expression of KLRG1 has not been studied in HIV-IBM before.

In this study, we aimed to characterize the clinical, morphological, and molecular features, including KLRG1 expression, of HIV-IBM to explore putative differences between HIV-associated IBM and sporadic IBM.

## Methods

### Patients

We identified 19 patients with myositis in the context of HIV infection. Seven of them fulfilled the ENMC criteria of clinicopathological defined IBM [21] and were included in the group of HIV-associated IBM. The remaining 12 patients were diagnosed with PM-Mito, IMNM-like pathology or non-specific myositis. Seven randomly selected IBM patients fulfilling the ENMC criteria for IBM were included in the group of so-called sporadic IBM. Biopsies from patients without pathological abnormalities and normal laboratory parameters were used as non-disease controls.

### Morphological analysis

Muscle biopsy specimens from participating partner sites were cryopreserved immediately after removal at − 80 °C before analysis.

8 µm cryostat sections were stained according to standard protocols, including H&E, Gomori trichrome, Elastica van Gieson, non-specific esterase, acid phosphatase, Kongo red, SDH and COX-SDH.

A Benchmark XT Immunostainer (Ventana Medical Systems, Illkirch, France) was used for immunohistochemical staining in a standardized manner. The primary antibodies are listed as follows (clone, dilution, company): mouse anti-human: CD8 (C8/114B, 1:100, DAKO), CD20 (L26, 1:400, DAKO), CD45 (UCHL1, 1:400, DAKO), CD56 (ERIC-1, 1:200, Serotec), CD68 (EBM11, 1:100, DAKO), C5b-9 (aE11, 1:100, DAKO), HLA-ABC/MHC cl. I (W6/32, 1:1000, DAKO), HLA-DR/MHC cl. II (CR3/43, 1:200, DAKO), MHCneo (NB-MHCn, 1:20, Novocastra), CD57 (SPM 129, 1:50, Zymed), PD1 (NAT105, 1:100, Abcam); rabbit anti-human: CD4 (SP35, 1:100, Zymed), CD3 (polyclonal, 1:100, DAKO), p62 (polyclonal, 1:100, Abcam), KLRG1 (polyclonal, 1:50, Proteintech), CD27 (EPR8569, 1:500, Abcam), PD-L1 (E1L3N, 1:100, Cell Signalling); rat anti-human: PD-L2 (TY25, 1:100, Abcam).

Fluorescence staining with luminescent conjugated oligothiophenes (LCO) and fluorescence double staining was performed in staining chambers after fixation in acetone for 10 min. For LCO staining, the sections were incubated with pFTAA for 30 min and afterwards washed with phosphate-buffered saline (PBS) and distilled water.

For double immunostaining, serum of the secondary antibody species was added first for 30 min to avoid non-specific binding. The incubation with the first primary antibody was at 4 °C overnight and was followed by the first fluorescent secondary antibody with an incubation time of 1 h at room temperature.

The protocol was repeated in the dark with a second primary antibody and a second fluorochrome-coupled secondary antibody on the same sections, each with an incubation time of 1 h. Each incubation was followed by a washing step with PBS for 2 × 5 min. Finally, the sections were aqueously mounted with Vectashield mounting medium with DAPI and stored at 4 °C.

Fluorochromes and other fluorescence staining solutions are listed as follows (dilution and company): goat anti-rabbit: AF488 (1:100, Invitrogen), Cy3 (1:100, Dianova); goat anti-mouse: AF488 (1:100, Invitrogen), Cy3 (1:100, Dianova); LCO pFTAA (1:500, Institute of Chemistry Linköping University, Sweden).

We used irrelevant antibody stains (either mouse/rabbit monoclonal isotype controls) as negative control, as well as omission of the primary antibodies. For positive controls, we used the respective tissues as mentioned by the companies.

### Semi-quantitative scores and overall severity score

To evaluate the expression of calibre variance and atrophy, connective/fat tissue proliferation, necrosis, regeneration, MHC cl.-I/MHC cl.-II expression, rimmed vacuoles, mitochondrial alterations, autophagy and cellular infiltration, a semi-quantitative score was used to differentiate between low, medium, and severe levels.

Mitochondrial accumulation was categorized into cap-like subsarcolemmal accumulations, subsarcolemmal accumulations surrounding the entire fibre, and “ragged blue” fibres. Autophagy was separated into singular fine-granular sarcoplasmic labelling by p62, presence within vacuoles in a few fibres and presence in vacuoles in many fibres. The stages of cellular infiltration were divided into 1–4 cells per high power field (HPF = 0.096 mm^2^), 4–20 cells per HPF and > 20 cells per HPF. Capillary pathology, complement activation and amyloid deposits were divided into present or absent. A severity level of 0–3 or 0–1 was assigned for each category, and the sum of these results in an overall severity score of 0–10. The results are illustrated in a heat map. For the quantitative counting of the cells, 10 HPF were averaged. The values are presented as boxplots with 1.5*IQR (Interquartile range) whiskers.

### RNA extraction and quantitative real-time PCR

According to the manufacturer's instructions, RNA was isolated using the triazole/chloroform method (Invitrogen, Carlsbad, CA, USA). The concentration of RNA and the degree of purity were measured with the Infinite M200 Microplate Reader (Tecan, Grödig, Austria, RRID: SCR_020543). Afterwards the complementary DNA was produced by reverse transcription using the High-Capacity cDNA Archive Kit (Applied Biosystems, Forster City, USA). To measure the expression level of the gene transcripts and an endogenous control gene (*PGK1*), quantitative PCR was performed using the 5' nuclease method on an Applied Biosystems™ QuantStudio™ 6 Flex Real‐Time PCR System (ThermoFischer, Waltham, MA; USA) with the following conditions: 95 °C 0:20, 95 °C 0:01, 60 °C 0:20, 45 cycles (values above 40 cycles were defined as not expressed). All genes were run as triplicates. The Ct values of the target genes were related to the endogenous control gene and Δ*C*_t_ (= *C*_t_—*C*_t_ of the endogenous control) was calculated.

The following Taq-Man Gene Expression Assays from Life Technologies/ThermoFisher were used: *B3GAT1 (CD57),* Hs01024500_m1; *CD244,* Hs00175569_m1; *CD27,* Hs00609654_g1; *KLRG1*, Hs00195153_m1; *PD1*, Hs01550088_m1; *PDL1*, Hs00204257_m1; *PDL2*, Hs00228839_m1; *PGK1*, Hs99999906_m1; *TBX21*, Hs00894392_m1.

### Statistical analysis and graphical illustration

ImageJ Version 1.53 (RRID: SCR_003070) was used for the image processing. GraphPad Prism 9.0.2 software (GraphPad Software, Inc, La Jolla, CA, RRID: SCR_002798) was used for the graphical illustration. The ∆CT values are shown on the inverse axis. Statistical calculations were performed for the Kruskal–Wallis test with Bonferroni–Dunn correction to analyse the quantitative differences of mRNA transcripts. The level of significance was set at 5% (*) and 1% (**).

## Results

### Clinical data

The clinical data for all patients are shown in Table 1. We included seven male patients with HIV-associated IBM, seven (six male) patients with sporadic IBM and six (five male) healthy controls. The mean age at HIV diagnosis was 40 years (SD = 10.5). The CD4^+^ nadir was between 34 and 320 cells/µl, but data were missing for four patients. HIV-associated coinfections were present in two patients with Hepatitis B. Complications of HIV infection included non-Hodgkin lymphoma, CMV pneumonia, and polyneuropathy. The mean time between HIV diagnosis and muscle biopsy was 12 years (SD = 7.5) and the majority (72%) received antiretroviral therapy at the time of biopsy. In one patient, the antiretroviral treatment was interrupted with a CD4^+^ count of 500 cells/µl at the biopsy, and data were missing from another patient. Some antiretroviral drugs like Zidovudine can cause myopathy with mitochondrial damage too [22], but (as far as data were available) there was no history of Zidovudine use in our patients. The mean age at biopsy was 53 years (SD = 4.7) in the HIV-IBM group, 70 years (SD = 4.3) in patients with sporadic IBM and 58 years (SD = 9.5) in healthy controls. On average, the onset of symptoms was 1.7 years (SD = 0.7) before the biopsy in HIV-IBM patients and 6.2 years (SD = 3.2) in the sIBM group. Patients with HIV-associated IBM developed symptoms significantly earlier (*p* < 0.01) and had a shorter period between the onset of symptoms and biopsy than patients with sporadic IBM. Other clinical parameters showed up almost comparable in both groups.

**Table 1:** Clinical data for the HIV-IBM, sIBM and healthy control group (NDC)

	HIV-IBM	sIBM	NDC
Sex			
♀	–	14% (*n* = 1)	17% (*n* = 1)
♂	100% (*n* = 7)	86% (*n* = 6)	83% (*n* = 5)
Age at IBM diagnosis/biopsy (years)			
Mean (± SD)	53 (± 4.7)	70 (± 4.3)	58 (± 9.5)
Onset of symptoms before biopsy (years)			
Mean (± SD)	1.7 (± 0.7)	6.2 (± 3.2)	
First symptom			
Myalgia	14% (*n* = 1)	–	
Weakness upper extemities	–	14% (*n* = 1)	
Weakness lower extremities	57% (*n* = 4)	57% (*n* = 4)	
Dysphagia	–	14% (*n* = 1)	
No data	29% (*n* = 2)	14% (*n* = 1)	
Distribution of weakness			
Distal > proximal upper extremity	14%	29%	
Proximal > distal lower extremity	57%	72%	
No data	43%	14%	
CK (U/l)			
Mean (± SD)	802 (± 241)	846 (± 289)	
Anti-cN1A			
Positive	–	29% (*n* = 2)	
Negative	57% (*n* = 4)	42% (*n* = 3)	
No data	43% (*n* = 3)	29% (*n* = 2)	
Drug therapy			
Steroids	–	–	
IVIG	42% (*n* = 3)	86% (*n* = 6)	
None	29% (*n* = 2)	–	
No data	29% (*n* = 2)	14% (*n* = 1)	
Treatment response (*incl. temporary)			
Yes*	67% (*n* = 2)	100% (*n* = 6)	
No	–	–	
No data	13% (*n* = 1)	–	
Age at HIV diagnosis			
Mean (± SD)	40 (± 10.5)		
CD4 nadir (CD4^+^ T cells/µl)			
< 200	29% (*n* = 2)		
200–500	14% (*n* = 1)		
> 500	–		
No data	57% (*n* = 4)		
Years between HIV diagnosis and biopsy			
Mean (± SD)	12 (± 7.5)		
ART at muscle biopsy			
Yes	72% (*n* = 5)		
No	14% (*n* = 1)		
No data	14% (*n* = 1)		
History of Zidovudine use			
Yes	–		
No	57% (*n* = 4)		
No data	43% (*n* = 3)		
HIV-associated co-infections			
Hepatitis B	29% (*n* = 2)		
Lues	–		
Chlamydia	–		
None	–		
No data	71% (*n* = 5)		
Other HIV-associated diseases (multiple answers possible)			
Malignoma	14% (*n* = 1)		
Opportunistic infections	14% (*n* = 1)		
Neuropathy	29% (*n* = 2)		
None	14% (*n* = 1)		
No data	57% (*n* = 4)		

Patients developed weakness of the thigh muscles as the most common first symptom. A general distribution of muscle weakness accentuated proximally in the lower extremities and mixed in the upper extremities. Of note, in two HIV-IBM patients, the muscle weakness in the upper extremities was more pronounced proximally. The mean creatine kinase (CK) serum levels were similar in both groups (see Table 1). The antibody anti-cN1A, non-specifically associated with IBM, was positive in two patients with sIBM but absent in all HIV-IBM patients. Most patients were treated with intravenous immunoglobulins (IVIG). As far as data were available, all patients (2 out of 3 HIV-IBM patients and six sIBM patients) showed at least subjective temporary improvement of muscle weakness and swallowing difficulties in patients affected by dysphagia (one patient from each group) after the first few months of IVIG treatment. Objectively, this improvement was confirmed with an increase in strength in two patients (one patient from each group) and a stabilisation of strength in another two sIBM patients in the clinical examination. A long-term course was available for three patients (two HIV-IBM patients and one sIBM patient): after 8–10 years of follow-up, clinical worsening was evident with initial MRC strength levels of 4–5, which deteriorated to MRC levels of 2–3. Of note, one sIBM patient also received corticosteroids and azathioprine, which did not improve symptoms, while this patient also temporarily benefited from IVIG.

### Morphological analysis of skeletal muscle tissues

**Fig. 1:**
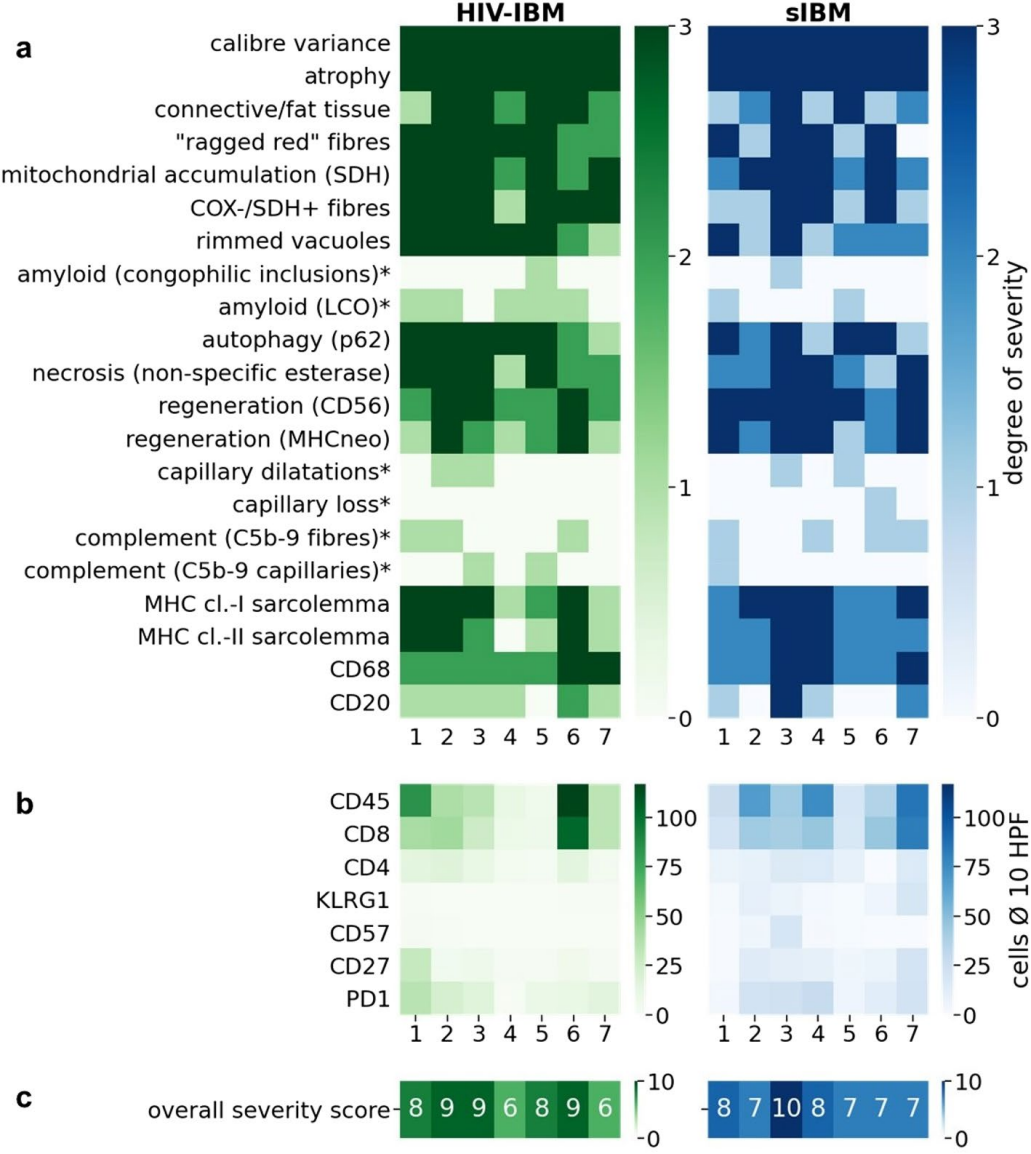
Heatmap: histomorphological features of patients with HIV-IBM (green) and sIBM (blue). In the upper part (**a**) all features are divided into degrees of severity from 0 to 3 or positive/negative (0–1) if marked with a star (*). The middle part (**b**) shows the average cell counts per HPF for immunohistochemistry of specific T cell markers. All features are summarized in the lower part of the heatmap (c) in an overall severity score from 0 to 10. The overall severity score averaged 8 out of 10 in both groups (**c**)

The histomorphological comparison revealed similar results in patients with HIV-associated IBM and sporadic IBM, with the following features being evaluated: mitochondrial damage, protein accumulation (rimmed vacuoles and amyloidogenic inclusions), autophagy, necrosis, capillary pathology, and inflammation markers (Fig. 1a). The mitochondrial damage with COX-negative fibres tended to be more severe in HIV-IBM patients. The presence of regenerating fibres with CD56 and MHC neonatal expression was more numerous in sIBM patients. Evidence of amyloid deposits was present in 71% of HIV-IBM patients and 43% of sIBM patients. The staining was conducted with LCOs and Congo red, whereby the LCOs proved to be more sensitive with a staining reaction in 5/7 patients of the HIV-IBM group and 2/7 sIBM patients. Congophilic inclusions were only detectable in one patient of each group. LCOs can adapt to the molecular structure of the deposited proteins and thereby detect a broader spectrum of amyloidogenic inclusions (57). All features are listed in Fig. 1a.

The extent of cellular infiltrates showed a wide range with 6 to 117 CD45 positive cells per HPF in the HIV-IBM group and 19 to 86 in the sIBM group, with predominantly more cytotoxic T-cells and a median number of 34 CD8^+^ cells per HPF (7–104 cells) in the HIV-IBM group and 43 (18–83 cells) in the sIBM group (Fig. 1 b). The final overall severity score averaged 8 out of 10 in both groups (Fig. 1 c).

T cell exhaustion and senescence.

**Fig. 2:**
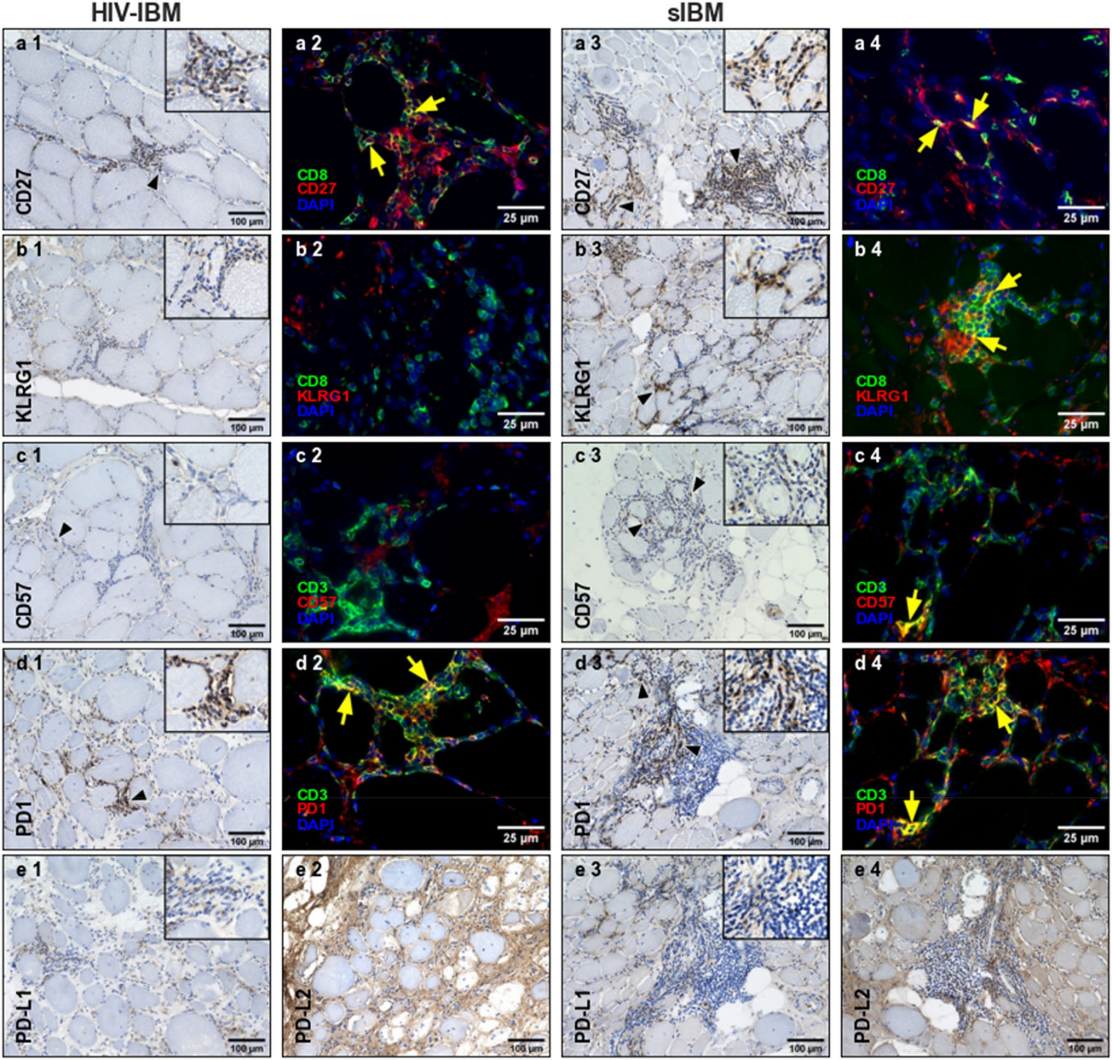
Immunohistochemistry and double immunofluorescence staining of HIV-IBM and sIBM. HIV-IBM cases showed no KLRG1^+^ or CD57^+^ cells in contrast to the sIBM cases (**b1–4**, **c1–4**). Many CD8^+^/CD27^+^ and CD3^+^/PD1^+^ cells were detectable in both groups (**a1–4**, **d1–4**). The ligands showed a weak expression for PD-L1 (**e1**, **e3**) and a non-specific staining pattern involving the endomysial connective tissue and myocyte sarcolemma cells for PD-L2 (**e2**, **e4**)

We detected a median value of five CD27^+^ cells per HPF (2–30 cells) in the HIV-IBM group and eight CD27^+^ cells per HPF (1–21 cells) in patients with sIBM (Figs. 2a1, a3, 3). KLRG1^+^ cells were mainly undetectable in the HIV-IBM group (0–1 cells) but showed a significantly (*p* < 0.01) higher expression in the sIBM group with a median value of six positive cells per HPF (1–19 cells) (Figs. 2b1, b3, 3). CD57 was almost not expressed in both groups, with a median value of zero cells per HPF (0–2 cells) in patients with HIV-IBM and one cell per HPF (0–19 cells) in the sIBM group. Only one sIBM patient showed a higher expression with 19 CD57^+^ cells per HPF (Figs. 2c1, c3, 3).

**Fig. 3:**
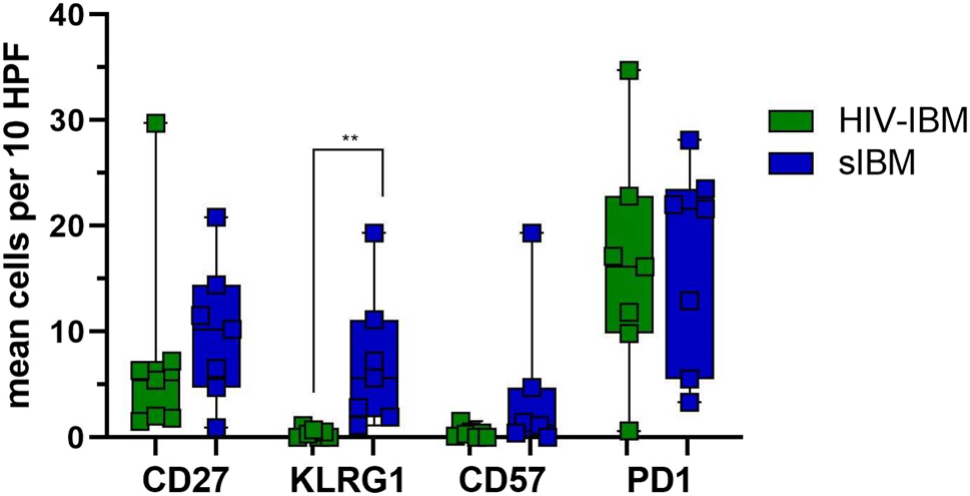
Quantitative immunohistochemistry for CD27, KLRG1, CD57 and PD1: comparison of HIV-IBM (green) and sIBM (blue). The average cell counts from ten high power fields (HPF) are shown in a boxplot. Statistical calculations were performed for the Mann–Whitney *U* test. The level of significance was set at 1% (**). KLRG1^+^ cells were undetectable in the HIV-IBM group, but showed a significantly higher expression in the sIBM group. There was no significant difference in the number of CD27-, CD57- and PD1-positive cells between patients with HIV-IBM and sIBM

We performed double immunofluorescence stains to allocate the markers to defined cell populations. In both groups, we were able to detect CD8^+^ cells with additional expression of CD27^+^ (Fig. 2a2, a4). The combination of CD8 and KLRG1 revealed a different result between the two groups. Several CD8^+^KLRG1^+^ cells were detected in sIBM patients but not in the HIV-IBM group (Fig. 2b2, b4). CD3 and CD57 were occasionally expressed in the same cells in the sIBM patients but not in the HIV-IBM group (Fig. 2c2, c4). Overall, the immunohistochemical findings were confirmed here: In patients with sporadic IBM, some CD8^+^ T cells also expressed KLRG1 and occasionally CD57. These cells were undetectable in patients with HIV-associated IBM.

The protein expression of the immune checkpoint molecule PD1 was similar in both groups, with a median value of 16 cells per HPF (1–35 cells) in the HIV-IBM group and 21 cells per HPF (3–28 cells) in the sIBM group (Figs. 2d1, d3; 3). Double immunofluorescence staining was performed to assign PD1 to the T cell population expressing the surface marker CD3. Numerous double-positive cells were detectable in both groups (Fig. 2d2, d4). PD-L1 staining showed a weak expression in both groups (Fig. 2e1, e3). PD-L2 staining revealed a non-specific pattern involving the endomysial connective tissue and myocyte sarcolemma cells. This was found in both groups, too (Fig. 2e2, e4).

**Fig. 4:**
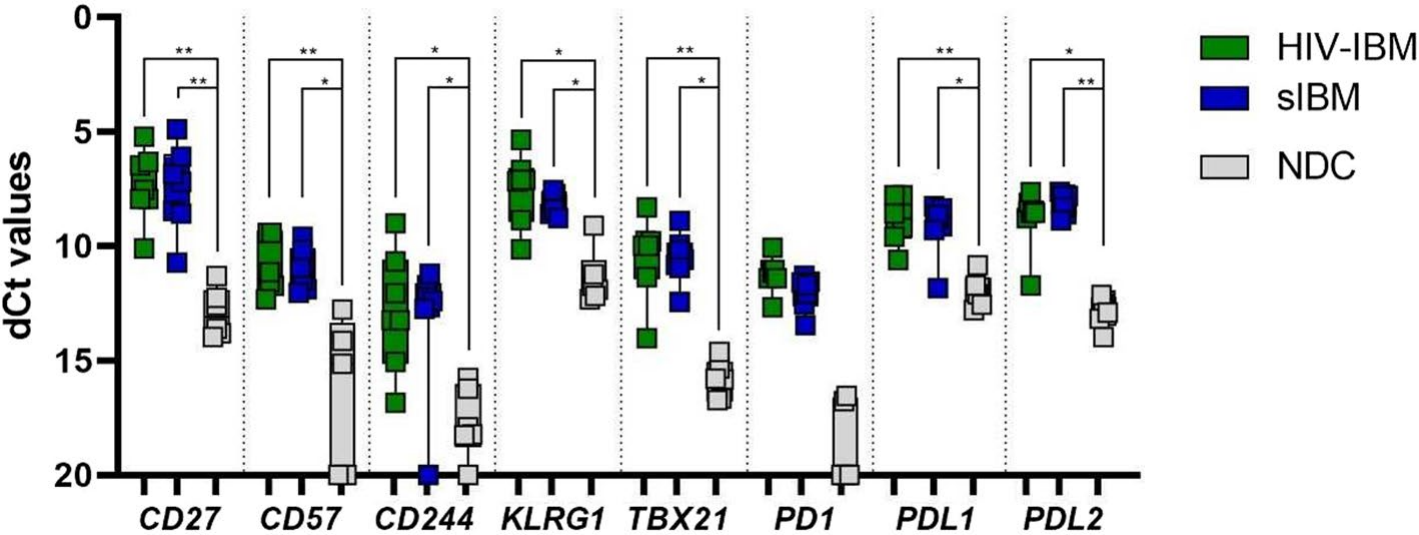
Quantitative PCR for CD27, CD57, CD244, KLRG1, TBX21, PD1, PDL1, PDL2: comparison of HIV-IBM (green), sIBM (blue) and NDC (grey). Delta CT values are shown in a boxplot on the inverse axis. Statistical calculations were performed for the Kruskal–Wallis test with Bonferroni–Dunn correction to analyze the quantitative differences of mRNA transcripts. The level of significance was set at 5% (*) and 1% (**). All markers were shown to be significantly upregulated at gene expression levels compared to the NDC group, with no significant difference between the IBM subgroups

Quantitative PCR was performed to measure the gene expression of immunoregulatory molecules (Fig. 4). The mRNA coding for the membrane receptors CD27, KLRG1, CD57, CD244 and PD1 with its ligands and for the transcription factor TBX21 were detected. The results showed no significant difference between the HIV-IBM and sIBM patients but a significant upregulation of all markers compared to the NDC group (Fig. 4). Since PD1 was below the detection limit in four healthy controls, a statistical calculation between the groups was impossible.

## Discussion

In this study, we present comprehensive clinical, histological, and molecular data comparing HIV-associated and sporadic IBM focussing on dysfunctional T cells. Overall, HIV-associated IBM and sporadic IBM share many clinical, morphological, and immunological features. However, critical clinical differences include significantly younger age at symptom onset and biopsy in HIV-IBM patients confirming the results of previous studies [3].

Despite a shorter disease duration, HIV-IBM patients showed the same degree of muscle damage in the overall severity score. This indicates that HIV serostatus may influence the development and course of IBM with a faster disease progression than HIV seronegative IBM patients. Since HIV patients in general receive special medical care, the increased attention and closer monitoring may have contributed to an earlier biopsy, too.

Of note, both patient groups showed subjective improvement of dysphagia and some temporary, self-reported beneficial effects on muscle strength after IVIG treatment with clinical worsening in the long-term course. While some previous studies have reported a possible temporary effect of IVIG treatment on dysphagia [23, 24], our data do not indicate a sustained and, importantly, objectively quantifiable impact on the course of the disease.

Differentiation stages and immunomodulatory molecules in cytotoxic T-cells were examined. We did not detect highly differentiated cytotoxic T-cells expressing KLRG1 and CD57 in the skeletal muscles of patients with HIV-IBM in contrast to patients with sIBM. Nevertheless, the proportion of KLRG1^+^ cells was also relatively low in the sIBM group, with a median of 6 from 43 CD8^+^ cells per HPF. At the same time, previous studies described 79% of CD8^+^ cells as KLRG1^+^ and 48% as CD57^+^ at the surface of muscle fibres in patients with a disease duration of 3–84 months [18]. Our results suggest that muscle-invading cytotoxic T cells in HIV-IBM may be at less advanced stages of differentiation with less expression of KLRG1 and CD57. Since these markers are associated with immune senescence, longer disease duration in the sIBM group could explain this difference. Recent studies have also shown a correlation between the disease duration and increased differentiation of CD8^+^ T cells in the blood of sIBM patients [19]. Furthermore, it has been demonstrated that KLRG1 expression is low in the early stages of IBM-SD (formerly called “PM-Mito”), but increasing with the development of histomorphological signs of full-blown sIBM [6]. Of note, early IBM-SD patients were older (mean age 65 years) at the time of biopsy than HIV-IBM patients in this cohort (mean age 53 years). However, there was a similar gene expression level of *KLRG1* and *CD57* in patients with HIV-associated and sporadic IBM, which was significantly increased in both groups compared to the NDC group. Further studies with more significant case numbers are needed to confirm the differences in markers of cytotoxic T-cell differentiation on the protein level. Since TEMRA cells are minimally or non-proliferative in the muscle of IBM patients, which has been discussed as a reason for the lack of response to classic immunosuppressants [15, 25], the differences in cytotoxic T-cell differentiation stages between HIV-associated and sporadic IBM might also be significant in the context of future treatment strategies. T-cells expressing PD1 were equally detectable in HIV-associated and sporadic IBM and accounted for the most significant proportion of cytotoxic T cells overall. Since exhausted T cells develop gradually from early differentiated T-cells, they may also be present in HIV-IBM patients with a shorter disease duration [12].

The strengths of this study are the combination of numerous clinical features of both HIV history and muscle-specific features, with extensive histomorphological and immunological characterisation. However, clinical data was lacking partially due to the study's retrospective nature and a limiting factor for comparing HIV-associated and sporadic IBM was the small number of cases, with only seven patients per group. Methods such as LCOs were used to detect amyloid deposits, which have so far been little used in similar studies. These proved to be more sensitive in IBM compared to conventional amyloid ligands such as Congo red.

In summary, our study offers relevant and new insights into HIV-associated IBM. It contributes to further understanding the pathogenesis of IBM and the peculiarities of the HIV-associated variant of the disease. Because of an altered immunological status due to the HIV infection and the clinical differences with a significantly younger age of onset of illness and a possible faster disease progression, patients with HIV-IBM should be highlighted as a particular subgroup of IBM in further studies, as the differences in differentiation stages of cytotoxic T cells could be relevant for possible new therapeutic approaches. KLRG1 + T cells are characteristic of sIBM but do not seem absolutely required for IBM to manifest.

## Supplementary Information

Below is the link to the electronic supplementary material.

Supplementary file1 (DOCX 18196 KB)

## Data Availability

The authors of this study state that they have full access to all data and take full responsibility for the analyses, the interpretation, and the conduct of the research. Data not provided in the article because of space limitations will be made available (anonymised) at the request of any qualified investigator for purposes of replicating procedures and results.

## Glossary

CK: Creatine kinase
COX: Cyclooxygenase
CT: Cycle threshold
DM: Dermatomyositis
ENMC: European Neuromuscular Centre
H&E: Hematoxylin and eosin
HPF: High power field
IMA: Isolated mitochondrial abnormalities
IMNM: Immune-mediated necrotizing myopathy
IQR: Interquartile range
IVIG: Intravenous immunoglobulins
KLRG1: Killer cell lectin-like receptor subfamily G member 1
LCO: Luminescent conjugated oligothiophenes
MHC: Major histocompatibility complex
MHC: Major histocompatibility complex
NDC: Non-disease control
PBS: Phosphate buffered saline
PD1: Programmed cell death protein 1
pFTAA: Pentameric formyl thiophene acetic acid
PGK1: Phosphoglycerate kinase 1
PM: Polymyositis
qPCR: Quantitative polymerase chain reaction
SDH: Succinate dehydrogenase
sIBM: Sporadic inclusion body myositis
TEMRA: Terminally differentiated effector memory T cells
TBX21: T-Box transcription factor 21
