# Supplementary material for: Morphological and molecular comparison of HIV-associated and sporadic inclusion body myositis
Source: J Neurol. 2023 Jun 6;270(9):4434–43. doi: 10.1007/s00415-023-11779-y (PMC10243696; doi:10.1007/s00415-023-11779-y)
Supplement: Supplementary file 1 — Supplementary file1 (DOCX 18196 KB) [file 415_2023_11779_MOESM1_ESM.docx]

**Supplementary data**

**Table 2:** Semiquantitative Score

| **Feature** | **0** | **1** | **2** | **3** |
| --- | --- | --- | --- | --- |
| - calibre variance, atrophy - connective/fat tissue - necrosis - regeneration - MHC cl.-I/II expression | none | low | medium | strong |
| - fibres with rimmes vacuoles - COX^-^/SDH^+^ Fibres | none | singular | few | multiple |
| - mitochondrial accumulation | none | „cap-like“ subsarcolemmal accumulation | subsarcolemmal accumulation edge the whole fibre („Pre-ragged-blue/red“) | „ragged- blue/red“- fibres |
| - autophagy | none | singular fine granular dyed fibres | concentrated within the vacuoles in a few fibres | concentrated within the vacuoles in multiple fibres |
| - cellular inflammation B-, T-cells and macrophages per HPF | none | 1-4 cells | 4-20 cells | cluster of more than 20 cells |
| - capillary dilatations - capillary loss - complement activation on fibres and capillaries - amyloid deposits (LCO, congophilic inclusions) | no | yes |  |  |


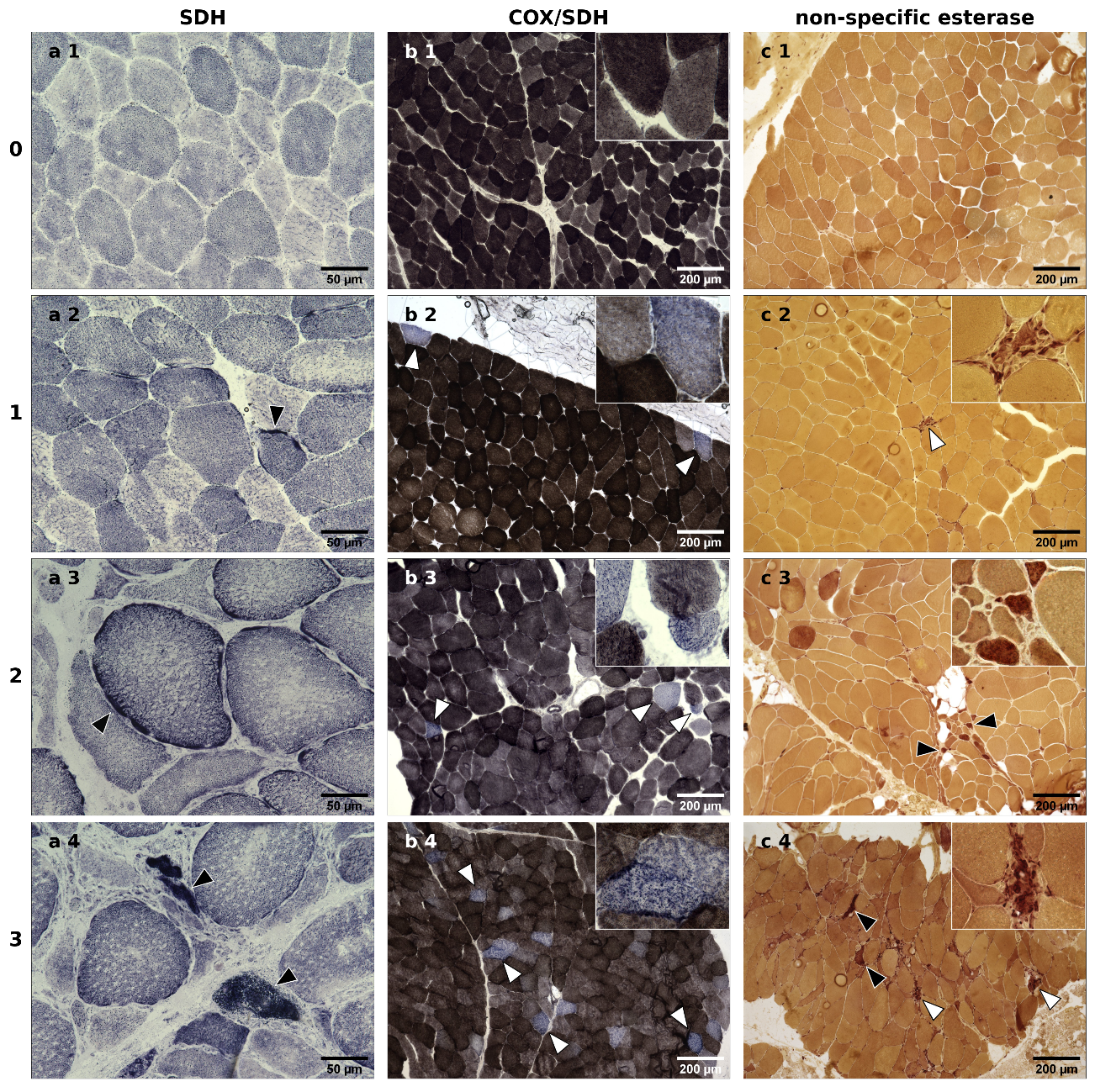


**Figure 5****: Severity of histopathological changes SDH, COX/SDH, non-specific esterase**

Severity levels of histopathological changes: 0 (none), 1 (mild), 2 (moderate), 3 (severe). SDH staining is divided into „cap-like“ subsarcolemmal accumulation (severity level 1, a2), “pre-ragged blue” fibres with subsarcolemmal accumulation around the whole fibre (severity level 2, a3) and “ragged blue” fibres (severity level 3, a4). The double staining with COX/SDH is divided into no, singular, few or multiple COX^-^/SDH^+^ fibres (b1-4). Staining of non-specific esterase shows necrosis (black arrows) and myophagocytosis (white arrows), which increase with each level of severity (c1-4).


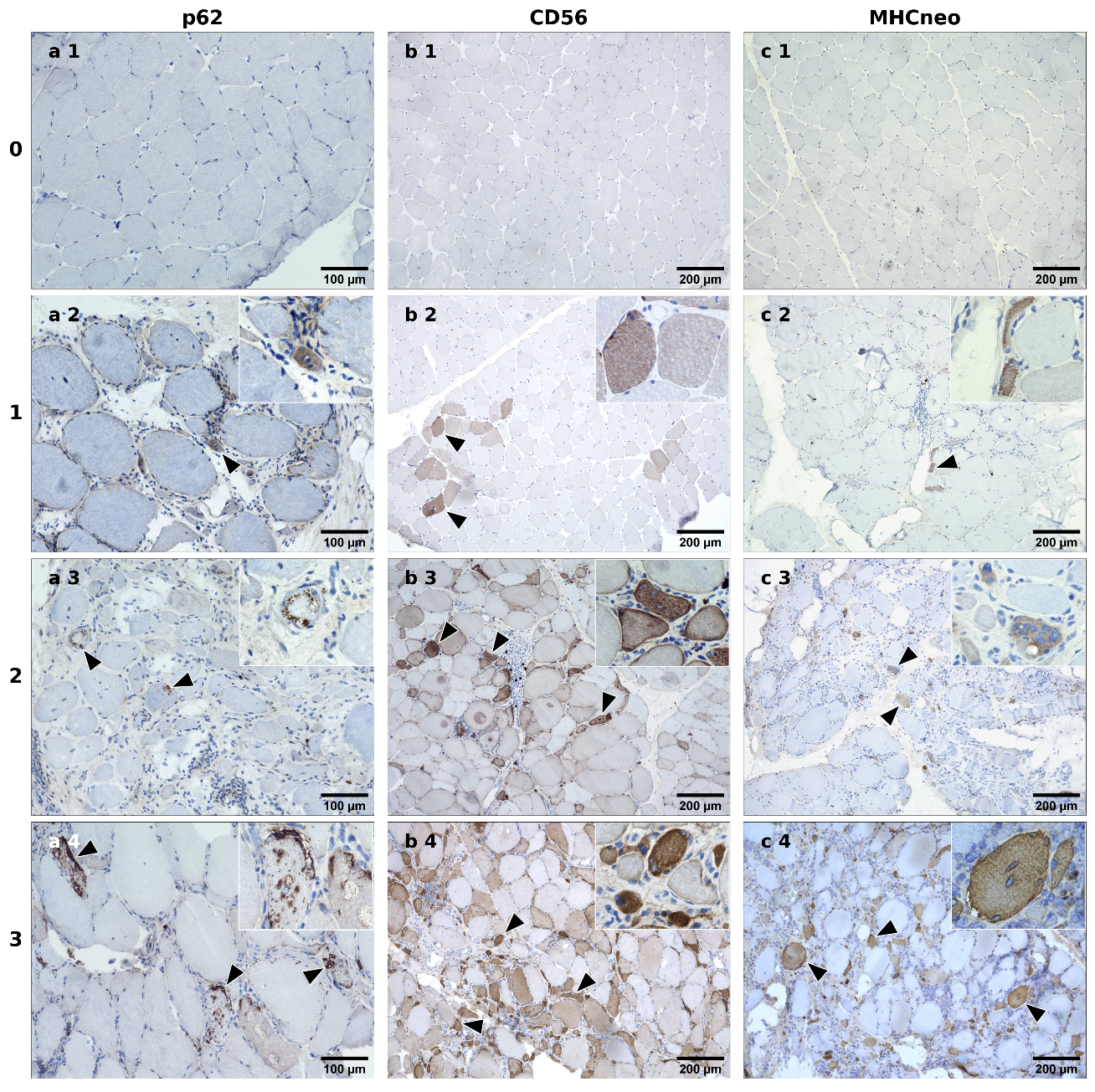


**Figure 6: Severity of histopathological changes p62, CD56, MHCneo**

Severity levels of histopathological changes: 0 (none), 1 (mild), 2 (moderate), 3 (severe). P62 staining shows autophagy with singular fine granular dyed fibres (severity level 1, a2), autophagic structures concentrated within the vacuoles in a few fibres (severity level 2, a3) and in multiple fibres (severity level 3, a4). CD56 and neonatal MHC staining show regenerating fibres with increasing numbers for each level of severity (b1-4, c1-4).


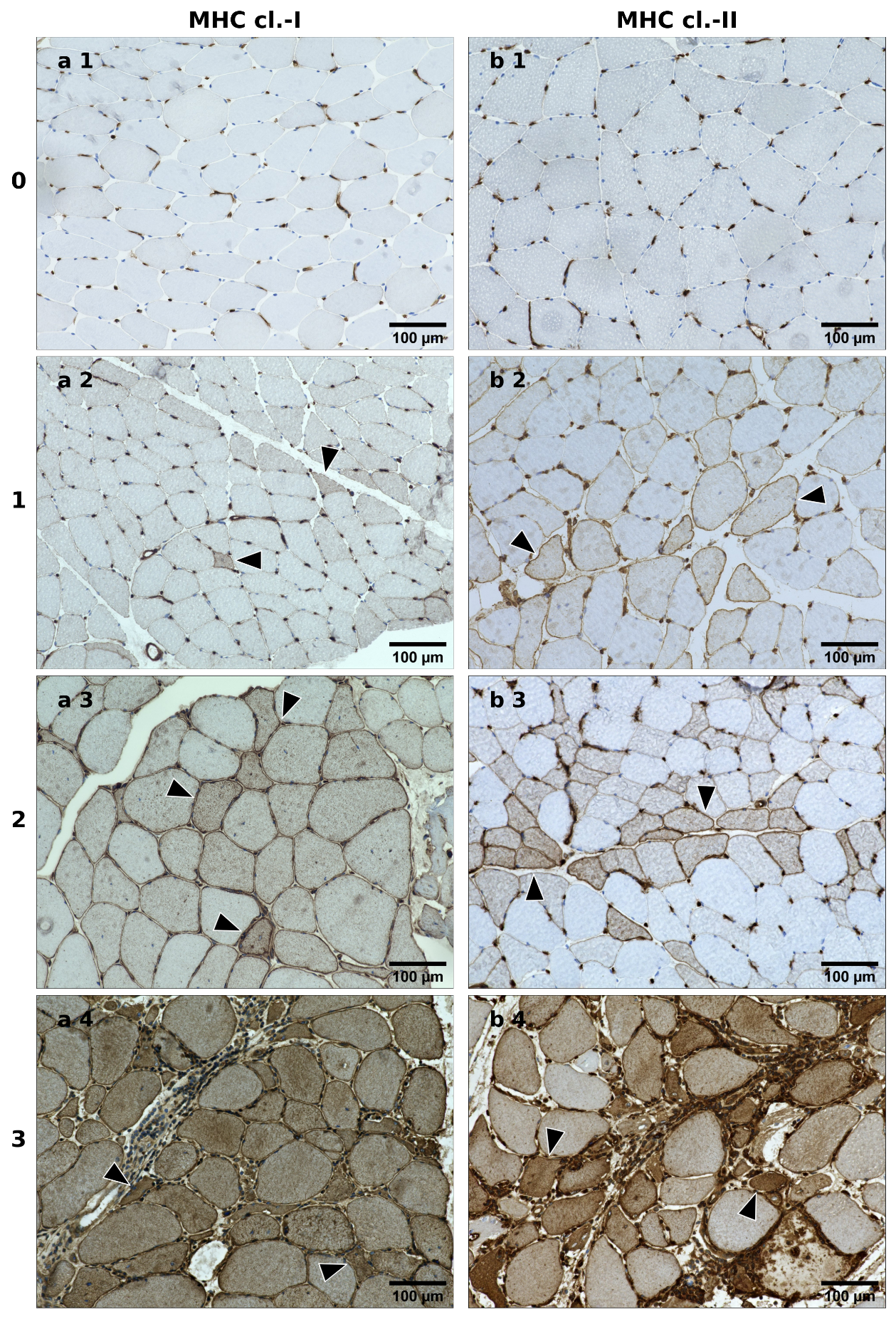


**Figure 7: Severtiy of histopathological changes MHC cl.-I und MHC cl.-II**

Severity levels of histopathological changes for MHC cl.-I (a1-4) and cl.-II (b1-4): 0 (none), 1 (mild), 2 (moderate), 3 (severe).


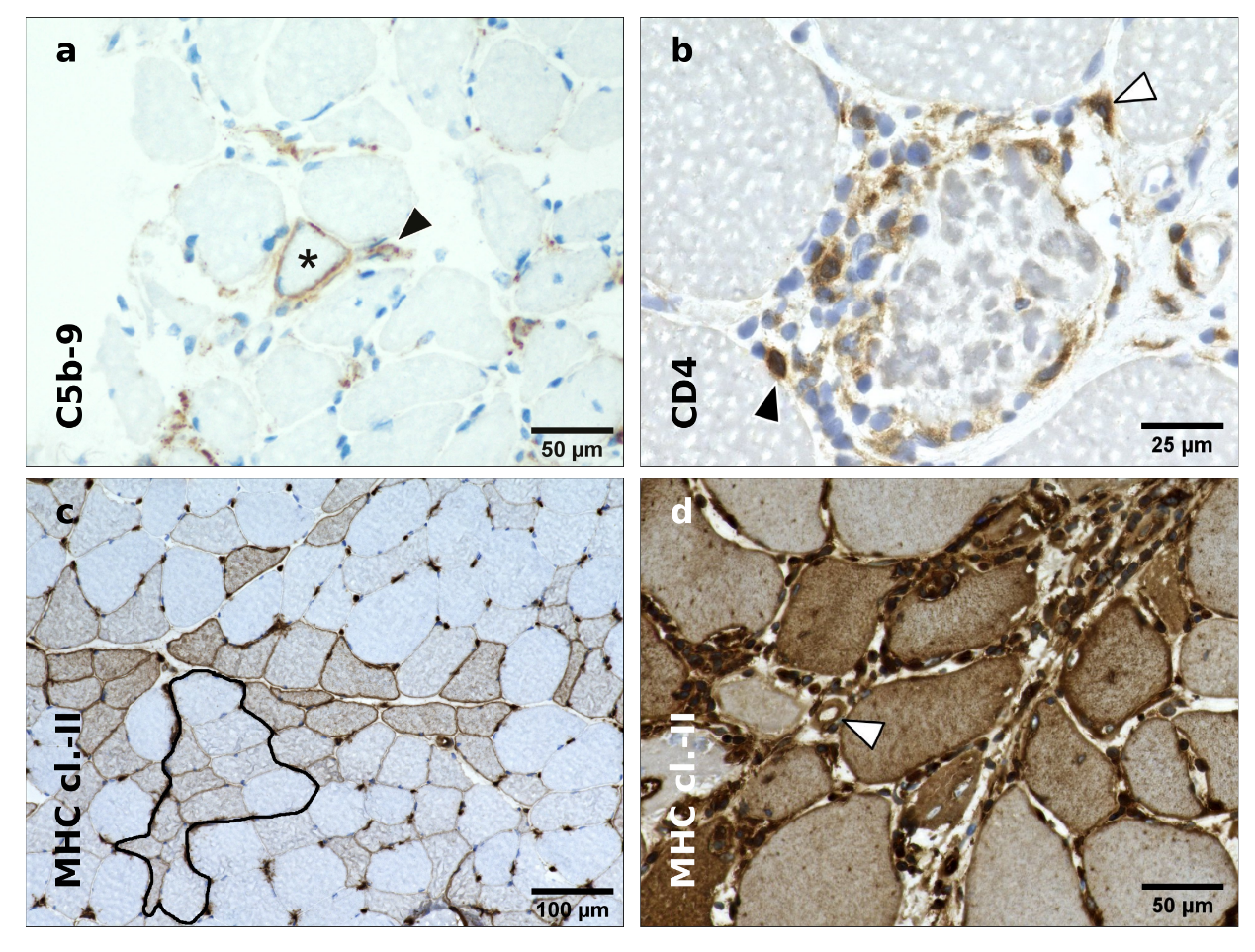


**Figure 8: Additional histological features**

Picture a shows sarcolemmal (black star) and capillary (black arrow) complement activation. The morphological differentiation of CD4 positive cells in lymphocytes (black arrow) and macrophages (white arrow) is shown in picture b. Capillary loss (area within the black border) and capillary dilatation (white arrow) are shown in picture c and d.


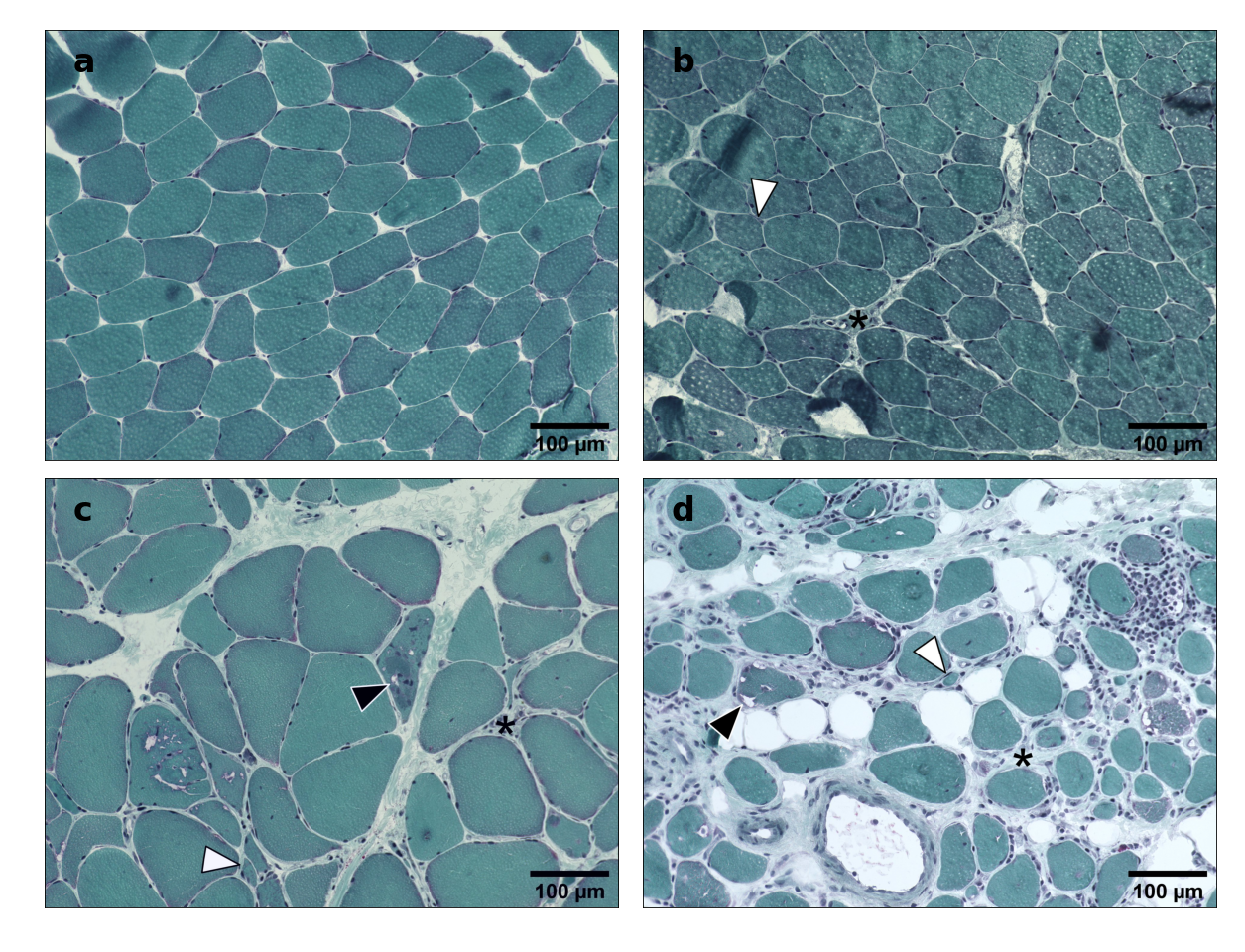


| **OSS** | **0** | **1** | **2** | **3** | **4** | **5** | **6** | **7** | **8** | **9** | **10** |
| --- | --- | --- | --- | --- | --- | --- | --- | --- | --- | --- | --- |
| **Score** | 0 | 1-6 | 7-12 | 13-18 | 19-24 | 25-30 | 31-36 | 37-42 | 43-48 | 49-54 | 55-60 |

**Figure 9: overall severity score**

The sum of all histological features forms the overall severity score with categories from 1-10, which was calculated for each patient. Examples for different severity levels are shown in Gomori trichrome staining: a) OSS 0; b) OSS 2; c) OSS 6; d) OSS 10. White arrows show atrophic fibres, black stars show endomysial proliferation of connective tissue and black arrows show rimmed vacuoles.
